# Otolaryngology-related symptoms of COVID-19 in children in the post-epidemic era: a cross-sectional web-based survey study

**DOI:** 10.3389/fped.2023.1190734

**Published:** 2023-08-04

**Authors:** Yong-chao Chen, Xin Wang, Yi-shu Teng, De-sheng Jia, Lan Li, Hong-guang Pan

**Affiliations:** ^1^ Department of Otorhinolaryngology, Shenzhen Children’s Hospital, Shenzhen, China; ^2^ Department of Otorhinolaryngology, Shenzhen Children’s Hospital, China Medical University, Shenzhen, China

**Keywords:** olfactory disorders, taste disorders, children, post-COVID-19, epidemic era

## Abstract

**Purpose:**

China adjusted and optimized its prevention and control strategies in December 2022, and it entered a new era of the coronavirus epidemic. Here, we describe the general and otolaryngology-related symptoms of coronavirus disease 2019 (COVID-19) in children during the first pandemic in the post-epidemic era, focusing on the frequency and severity of smell and taste loss, as well as the recovery process and its influencing factors.

**Patients and methods:**

From 2 January to 7 January 2023, we conducted a cross-sectional online questionnaire survey through Questionnaire Stars in order to collect relevant information about COVID-19 in children in Shenzhen.

**Results:**

A total of 1,247 valid questionnaires were received, with an effective response rate of 78.72%. All of the diagnoses were confirmed by nucleic acid or antigen test for COVID-19. Among the subjects, the sex ratio of male to female was more inclined to male (1.35:1), and the age was 3–16 years, with an average of 10.13 ± 2.82 years old. The most common symptoms were cough (58.24%), stuffy nose (56.18%), headache (42.09%), fatigue (40.44%), and sore throat (31.63%). Approximately 6.43% of the children reported dysosmia, the mean time of the duration of dysosmia was 5.38 ± 2.92 days, and the mean score of the severity of the dysosmia as assessed by visual analogue scale (VAS) was 4.63 ± 2.29. Approximately 13.34% reported dysgeusia, the mean time of the duration of dysgeusia was 4.77 ± 3.98 days, and the mean score of the severity of the dysgeusia as assessed by VAS was 5.12 ± 2.29. Univariate and multivariate logistic regression analysis showed that the prevalence of taste and olfactory disorders increased with age, mainly in children with severe symptoms and older children.

**Conclusion:**

In the post-epidemic era, due to weakening of the pathogenicity of the subvariant of Omicron, overall condition of children with COVID-19 was mild, incidence of olfactory and taste disorders was low, recovery was faster, and prognosis was better. In our study, cough, runny nose, and sore throat were the most common symptoms, and the prevalence of taste and olfactory disorders increased with age, mainly in older children with severe symptoms.

## 1. Introduction

COVID-19 is a severe respiratory syndrome generated by an infection with SARS-CoV-2 that can lead to serious complications and death, and at present, it is one of the most severe public health emergencies throughout the world (1). Globally, as of 13 January 2023, the pandemic has resulted in approximately 661,545,258 confirmed cases of COVID-19, including 6,700,519 deaths, reported to the WHO (2). Reported illnesses have ranged from mild symptoms to severe illness for COVID-19 cases (3, 4). Under the ongoing pandemic situation, pediatric cases are showing an increasing trend in many countries of the world (5). However, it is worth noting that the COVID-19 pandemic affects all age groups of children and appears to be a mild or moderate illness, which can include fever and cough (5).

In China, the Chinese government established a prevention and control policy involving restricting the flow of people and vigorously publicizing since the outbreak in Hubei, China, in January 2020 (6). To date, substantial research data have indicated that China’s prevention and control of COVID-19 epidemic are beginning to bear fruit (7, 8). Currently, with mass vaccination and mutation of new coronaviruses, the virus has become more transmissible but less pathogenic, and the overall health risk to the population tends to moderate. China is facing a new situation and new tasks in epidemic prevention and control. On 7 December 2022, China released a circular on further optimizing the COVID-19 response, announcing 10 prevention and control measures (9). Since this time, Omicron rapidly spread throughout China, and Omicron cases have peaked in China. China entered the era of post-novel coronavirus epidemic.

It is well known that the symptoms of COVID-19 are variable, and the age, comorbidities, and the virus variant are of great importance here. Regardless of the prevailing symptoms in the subsequent waves of infection, problems with the nose, throat, or ear were quite frequent. Here, we describe the general and otolaryngology-related symptoms of COVID-19 in children during the first pandemic in the post-epidemic era, focusing on the frequency and severity of smell and taste dysfunction, as well as the recovery process and its influencing factors.

However, survey-type studies are not ideal to calculate the prevalence of conditions, because those people with more symptoms, or with more pronounced symptoms, might have felt more motivated to participate in the survey, while in the absence of such symptoms, people infected with COVID-19 are less likely to respond. This would overestimate the overall frequency of the symptoms, because the survey mechanism concentrates subjects with such symptoms.

## 2. Patients and methods

### 2.1. Study design and population

This is a cross-sectional online questionnaire study, an online questionnaire that collects information from pediatric patients (aged 1–16 years) who had a history of COVID-19 infection in Shenzhen, China. The questionnaire was administered using Questionnaire Star (www.wjx.cn) and was administered from 2 January to 7 January 2023. The questionnaire link and survey information were sent out by social media of WeChat, and data were retrieved by a background database. Only children confirmed to have COVID-19 by performing real-time reverse transcription–polymerase chain reaction (RT–PCR) test or rapid antigen detection test were eligible to participate. Each WeChat account could complete the questionnaire only once. Informed consent was obtained at the start of the online questionnaire.

### 2.2. Structured questionnaires

The questionnaire contained 26 questions which consisted of general questions including age, sex, smoking history, and concomitant diseases, presenting symptoms, and questions about the severity and duration of otolaryngology-related symptoms during illness. In the case of loss of sense of smell, it was asked whether the patients also have parosmia (i.e., distorted perception of odor with a known source) or odor phantoms (i.e., odor sensation without an odor source). In the case of loss of sense of taste, subjects were asked to answer further questions regarding taste characteristic. The severity of otolaryngology-related symptoms during illness was analyzed using visual analogue scale (VAS) scores normalized to a 10-point scale, the most severe symptoms were scored as 0 points, and no symptoms—10 points. It was assumed that the range of points 0–3 referred to severe symptoms, 4–6—moderate, and 7–10—mild.

### 2.3. Statistical analysis

The experimental data were analyzed by SPSS 26.0 statistical software. All the analyses were bilateral tests, and the test level was *α* = 0.05. The difference was statistically significant. The measurement data were expressed as mean ± SD, and the counting data were expressed as examples (percentage). When the measurement data between the two groups had a normal distribution, *t*-test was used (unpaired samples used unpaired *t*-test, and paired samples used paired *t*-test), and non-parametric rank sum test (unpaired samples used Mann–Whitney *U* test, and paired samples used Wilcoxon test) was used when they were not. Chi-square test was used to compare the numerical data between groups. To explore the association between taste and smell abnormalities and demographic and clinical characteristics, we used a univariate and multivariable adjusted logistic regression model.

## 3. Results

### 3.1. Participants’ characteristics

A total of 1,584 questionnaires were distributed, 1,247 COVID-19 confirmed case questionnaires were included in this study, and 337 questionnaires were excluded due to a large amount of incomplete data or children who were not tested for COVID-19. From the questionnaire, we see that the gender ratio of male to female was more inclined to male (1.35:1). The mean age of the sample was 10.13 ± 2.82 years, ranging from 3 to 16 years. The participants were grouped in terms of age, and they were divided into 3- to 5-year-old group (71, 5.85%), 6- to 8-year-old group (277, 22.82%), 9- to 11-year-old group (416, 34.27%), and 12- to 16-year-old age group (450, 37.07%). The percentage of children exposed to smoking or second-hand smoke was 5.27% (64/1,214). There was no statistical difference in terms of gender and the prevalence of exposure to smoking or second-hand smoke between groups (*p* > 0.05). In the investigated population, 42 (3.46%) had not received varicella vaccine, 27 (2.22%) had received one dose, 978 (80.56%) had received two doses, 165 (13.59%) had received three doses, and two (0.16%) had received four doses of the vaccine. The main findings are described in Table 1.

**Table 1:** The characteristic of COVID-19 patients in children (*n* = 1,214).

Characteristic	3–5 (*n *=* *71)	6–8 (*n *=* *277)	9–11 (*n *=* *416)	12–16 (*n *=* *450)	Overall (*n *=* *1,214)	*P*
1. Gender						
Boys	42 (59.15%)	168 (60.65%)	235 (56.49%)	252 (56.00%)	703 (57.91%)	0.256
Girls	29 (40.85%)	109 (39.35%)	181 (31.49%)	198 (44.00%)	522 (43.00%)	
2. Smoking/second-hand smoke exposure	5 (7.04%)	20 (7.22%)	22 (5.29%)	28 (4.82%)	64 (5.27%)	0.658
3. COVID-19 vaccination status						
Not vaccinated	16 (22.54%)	8 (2.89%)	11 (2.64%)	7 (1.56%)	42 (3.46%)	0.000
First	10 (14.08%)	4 (1.44%)	5 (1.20%)	8 (1.78%)	27 (2.22%)	
Second	37 (52.11%)	237 (85.56%)	342 (82.21%)	362 (80.44%)	978 (80.56%)	
Third	8 (11.27%)	28 (10.11%)	58 (13.94%)	71 (15.78%)	165 (13.59%)	
Fourth	0 (0.00%)	0 (0.00%)	0 (0.00%)	2 (0.44%)	2 (0.16%)	
4. General symptoms						
Fever	7 (9.86%)	53 (19.13%)	77 (18.51%)	87 (19.33%)	224 (18.45%)	0.345
Cough	38 (53.52%)	126 (45.49%)	244 (58.65%)	299 (66.44%)	707 (58.24%)	0.000
Headache	11 (15.49%)	93 (33.57%)	171 (41.11%)	236 (52.44%)	511 (42.09%)	0.000
Chest tightness	0 (0.00%)	5 (1.81%)	7 (1.68%)	27 (6.00%)	39 (3.21%)	0.000
Asthenia	15 (21.13%)	87 (31.41%)	157 (37.74%)	232 (51.56%)	491 (40.44%)	0.000
Muscle pain	7 (9.86%)	32 (11.55%)	71 (17.07%)	116 (25.78%)	226 (18.62%)	0.000
Nausea or vomiting	6 (8.45%)	37 (13.36%)	53 (12.74%)	63 (14.00%)	159 (13.10%)	0.408
Diarrhea or abdominal pain	4 (5.63%)	23 (8.30%)	25 (6.01%)	33 (7.33%)	84 (6.92%)	0.974
5. Otolaryngology-related symptoms						
Runny/stuffy nose	42 (59.15%)	138 (49.82%)	210 (50.48%)	292 (64.89%)	682 (56.18%)	0.000
Sore throat	8 (11.27%)	50 (18.05%)	130 (31.25%)	196 (43.56%)	384 (31.63%)	0.000
Epistaxis	3 (4.23%)	20 (7.22%)	15 (3.61%)	23 (5.11%)	61 (5.02%)	0.515
Snoring	11 (15.50%)	25 (9.03%)	25 (6.01%)	38 (8.44%)	99 (8.15%)	0.346
Loss of sense of smell	2 (2.82%)	5 (1.81%)	21 (5.05%)	50 (11.11%)	78 (6.43%)	0.000
Loss of sense of taste	3 (4.23%)	21 (7.58%)	50 (12.02%)	88 (19.56%)	162 (13.34%)	0.000
Hearing loss	0 (0.00%)	1 (0.36%)	4 (0.96%)	11 (2.44%)	16 (1.32%)	0.006
Ear pain	4 (5.63%)	2 (0.72%)	8 (1.92%)	21 (4.67%)	35 (2.88%)	0.019

### 3.2. General and otolaryngology-related symptoms

**Figure 1:**
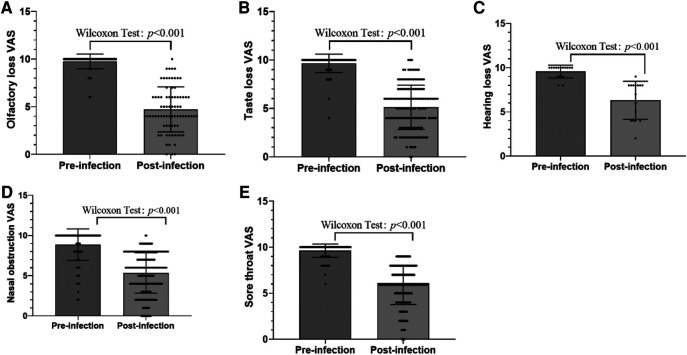
(**A**) Severity of olfactory loss assessed on the VAS (1–10). (**B**) Severity of taste loss assessed on the VAS (1–10). (**C**) Severity of hearing loss assessed on the VAS (1–10). (**D**) Severity of nasal obstruction assessed on the VAS (1–10). (**E**) Severity of sore throat assessed on the VAS (1–10).

The frequency of general and otolaryngology-related symptoms during COVID-19 disease is shown in Table 1. The most general symptoms were cough (58.24%), headache (42.09%), asthenia (40.44%), muscle pain (18.62%), and fever (18.45%). The frequency of otolaryngology-related symptoms in the order from the highest to lowest were runny/stuffy nose (56.18%), sore throat (31.63%), loss of sense of taste (13.34%), snoring (8.15%), loss of sense of smell (6.43%), epistaxis (5.02%), ear pain (2.88%), and hearing loss (1.32%). The younger the age, the less severe the COVID-19 disease. Among those exposed to smoking or second-hand smoke, the frequency of general and otolaryngology-related symptoms was higher. The severity of otolaryngology-related symptoms assessed on the VAS (1–10) is shown in Figure 1. Patient symptom severity scores revealed that the patients displayed mild-to-moderate symptom severity (olfactory, 4.63 ± 2.29; taste, 5.12 ± 2.29; hearing, 7.2 ± 1.92; nasal obstruction, 5.35 ± 2.51; sore throat, 5.87 ± 2.1). Additionally, 4.8% were asymptomatic cases.

### 3.3. Loss of sense of smell

A total of 78 respondents (6.43% of total; 40 girls, 51.28%) reported that they had loss of sense of smell in the course of infection. No parosmia or phantom smell was reported. A total of 13 respondents (16.66%) had loss of sense of smell on the day of their diagnosis, and 15 respondents (19.23%) with loss of sense of smell had neither runny/stuffy nose nor epistaxis. The average beginning of loss of sense of smell after diagnosis was 2.1 ± 1.84 days after diagnosis. The mean duration of smell disturbance was 5.38 ± 2.92 days, and 16 (20.51%) patients with loss of sense of smell had not fully recovered at the time of completing the questionnaire. Among them, loss of sense of smell was mild in 15 (19.23%) patients, moderate in 41 (51.28%), and severe in 22 (30.2%), the average severity of which was 4.63 ± 2.29 estimated on the VAS. The mean VAS of smell symptoms before and after infection is shown in Figure 1A. We then considered patients without olfactory dysfunction as controls, deriving an odds ratio (OR) to assess differences in the prevalence of various factors by univariate and multivariate analyses (Table 2). Based on the results of the univariate analysis, the factors that were significantly related to positive results (*P*-value of <0.05) were age, cough, headache, runny/stuffy nose, sore throat, and asthenia. The rate of personal history of allergic rhinitis was higher in the loss of sense of taste group than the no loss of sense of taste group, but the difference was not statistically significant [OR: 0.636, 95% confidence interval (CI): 0.374–1.081, *P* = 0.095]. Furthermore, multiple regression analysis of these factors showed that individuals with older age, cough, headache, sore throat, and asthenia were significantly higher prevalent.

**Table 2:** Univariate and multivariate analysis in patients with loss of sense of smell.

Factor	Univariate analysis	Multivariate analysis		
	OR	*P*	OR	*P*
Gender (male vs. female)	1.454 (0.919–2.302)	0.110	–	
Age (years old)	1.267 (1.151–1.395)	0.000	1.145 (1.034–1.267)	0.009
Smoking/second-hand smoke exposure (yes vs. no)	1.005 (0.355–2.843)	0.993	–	–
Vaccination history (yes vs. no)	1.387 (0.329–5.848)	0.656	–	–
≥3 doses	1.405 (0.769–2.568)	0.269	–	–
Personal history of allergic rhinitis (yes vs. no)	0.636 (0.374–1.081)	0.095	0.678 (0.390–1.182)	0.171
Fever (yes vs. no)	1.790 (0.880–3.641)	0.108	–	–
Cough (yes vs. no)	4.247 (2.271–7.944)	0.000	2.131 (1.076–4.220)	0.030
Headache (yes vs. no)	3.566 (2.161–5.885)	0.000	1.891 (1.099–3.251)	0.021
Runny/stuffy nose (yes vs. no)	2.976 (1.717–5.158)	0.000	1.388 (0.759–2.583)	0.287
Sore throat (yes vs. no)	4.843 (2.974–7.889)	0.000	2.270 (1.317–3.911)	0.003
Asthenia (yes vs. no)	3.839 (2.326–6.337)	0.000	1.859 (1.071–3.225)	0.027

OR, odds ratio.

The *P* value represents the *p* value of the univariate analysis.

### 3.4. Loss of sense of taste

A total of 162 respondents (13.34% of total; 68 girls, 41.96%) reported that they had loss of sense of taste in the course of infection. Only 21 respondents (12.96%) had loss of sense of taste on the day of their diagnosis. The average beginning of loss of sense of taste after diagnosis was 2.61 ± 2.57 days after diagnosis. Only 62 respondents (38.27%) described loss of the senses of both smell and taste together. Among them, the loss of sense of taste appeared after (*n* = 10, 16.13%), before (*n* = 9, 14.52%), or at the same time as the appearance of the loss of sense of smell (*n* = 43, 69.35%). The mean duration of taste disturbance was 4.77 ± 3.98 days, and 22 (13.58%) patients with loss of sense of taste showed not fully recovered at the time of completing the questionnaire. Among them, loss of sense of taste was mild in 44 (27.16%) patients, moderate in 78 (48.15%), and severe in 40 (24.69%), the average severity of which was 5.12 ± 2.29 estimated on the VAS. The mean VAS of taste symptoms before and after infection is shown in Figure 1B. We then considered patients without dysgeusia as controls, deriving an OR to assess differences in the prevalence of various factors by univariate and multivariate analyses (Table 3). Based on the results of the univariate analysis, the factors that were significantly related to positive results (*P*-value of <0.05) were age, personal history of allergic rhinitis, fever, cough, headache, runny/stuffy nose, sore throat, and asthenia. Furthermore, multiple regression analysis of these factors showed that individuals with older age, headache, sore throat, and asthenia were significantly higher prevalent.

**Table 3:** Univariate and multivariate analysis in patients with loss of taste.

Factor	Univariate analysis		Multivariate analysis	
	OR	*P*	OR	*P*
Gender (male vs. female)	0.972 (0.695–1.358)	0.866	–	–
Age (years old)	1.196 (1.120–1.277)	0.000	1.110 (1.035–1.191)	0.004
Smoking/Second-hand smoke exposure (yes vs. no)	0.434 (0.155–1.212)	0.111	–	–
Vaccination history (yes vs. no)	1.145 (0.443–2.956)	0.780	–	–
≥3 dose	0.983 (0.607–1.592)	0.944	–	–
Personal history of allergic rhinitis (yes vs. no)	0.651 (0.447–0.949)	0.026	0.683 (0.460–1.013)	0.058
Fever (yes vs. no)	2.637 (1.493–4.655)	0.001	1.710 (0.938–3.115)	0.080
Cough (yes vs. no)	2.036 (1.414–2.932)	0.000	1.127 (0.743–1.709)	0.574
Headache (yes vs. no)	2.593 (1.843–3.647)	0.000	1.495 (1.025–2.178)	0.037
Runny/stuffy nose (yes vs. no)	2.165 (1.507–3.111)	0.000	1.337 (0.892–2.002)	0.159
Sore throat (yes vs. no)	3.320 (2.367–4.657)	0.000	1.910 (1.297–2.813)	0.001
Asthenia (yes vs. no)	3.279 (2.317–4.641)	0.000	1.843 (1.249–2.721)	0.002

OR, odds ratio.

## 4. Discussion

Before December 2022, China has adopted a different response strategy from other countries. From the closure of Wuhan in 2020 to the dynamic zero COVID policy, precise prevention and control has prevented the spread of the new coronavirus (6). With the weakening of the pathogenicity of COVID-19, the popularization of vaccination, and the accumulation of experience in prevention and control, China had adjusted and optimized its prevention and control strategies in December 2022 (9). After China adjusted its prevention and control policy, the rapid spread of the epidemic in China has caused a worldwide concern. As a special group, children have immature cellular and humoral immune functions and are vulnerable to viral infections. Here, we investigated the frequency and severity of otolaryngology-related and general symptoms in pediatric patients who were confirmed COVID-19 and the recovery process of smell and taste during the first pandemic in the post-epidemic era.

Omicron is extremely contagious compared with previous variants, but COVID-19 disease symptoms were often not visible in children, and the younger the age, the less severe the disease. Omicron variant is often confined to the upper respiratory tract such as the nose, the throat, and the respiratory tract, which causes less damage to the lungs compared with other variants. During this Omicron pandemic, the most common clinical features in pediatrics are cough and runny/stuffy nose and reported in 58.24% and 56.18%, respectively in our study. Jichao Sha et al. (10) retrospectively analyzed the clinical and upper airway characteristics of 3,715 patients (range 2–86 years) with the Omicron variant, and they found that the main clinical characteristics of the SARS-CoV-2 Omicron variant are upper airway symptoms and general symptoms, while fever remains the most common symptom, followed by mild dry cough. Previous studies reported such symptoms as fever, cough, vomiting, and breathing difficulties as the most clinical manifestations of the Omicron variant in children (11, 12). However, our results showed that the proportion of patients with fever or gastrointestinal or upper respiratory symptoms was not high, possibly due to the younger age and lower rate of symptoms, and some may only exhibit cough or runny/stuffy nose. The relatively mild condition of children after infection may be related to the low functional expression or relatively weak expression of ACE2 receptor, and the pathway of virus invasion is limited, which avoids the possibility of severe occurrence after infection in children (13).

A larger proportion of asymptomatic infections with omicron are reported. Based on meta-analyses of published evidence on COVID-19 Omicron wave, Yu et al. (14) showed the proportion of asymptomatic infection to be 21.4% (14.5%–31.6%), 82.5% (78.1%–87.2%), and 62.0% (61.3%–62.6%) in studies from all aged group, children and adolescent group, and elderly group, respectively. Studies by Li et al. (15) showed that the proportion of asymptomatic people (adults and children) with Omicron in early 2022 was 23.5% in Shenzhen. In 2022, Li et al. (16) reported that studies in children revealed up to 83% asymptomatic infections. However, the asymptomatic case rate in our study was much lower (4.8%), which further indicated that some asymptomatic SARS-CoV-2-infected individuals were not willing to answer the questionnaire, and the overall frequency of the symptoms might be overestimated.

Taste and smell dysfunctions were common manifestations in COVID-19 adults prior to Omicron (17–19). In 2020, a meta-analysis of 104 studies, including 38,198 patients with COVID-19, showed that estimated random prevalence of olfactory dysfunction was 43.0%, that of taste dysfunction was 44.6%, and that of overall chemosensory dysfunction was 47.4% (20). In 2021, a systematic review and meta-analysis by Mutiawati et al. (21) showed that out of 32,142 COVID-19 patients from 107 studies, anosmia was reported in 12,038 patients with a prevalence of 38.2% (95% CI: 36.5%, 47.2%); whereas dysgeusia was reported in 11,337 patients out of 30,901 COVID-19 patients from 101 studies, with a prevalence of 36.6% (95% CI: 35.2%, 45.2%), worldwide. However, the Omicron variant has been reported to cause less taste and smell dysfunction than the preceding SARS-CoV-2 virus variants (22). von Bartheld et al. (22) recently published a meta-analysis and systematic review showing that the global prevalence of Omicron-induced olfactory dysfunction in adults is 3.7%, and the prevalence of olfactory dysfunction after omicron infection is about 2–10-fold lower than with previous variants. Additionally, Zhang et al. (8) showed that loss of smell and taste (1.3%) was also much reduced with Omicron in Shenzhen.

A lower prevalence of smell dysfunction (or chemosensory dysfunction) has been detected in children. In the pre-Omicron era, a meta-analysis of the prevalence of smell and taste dysfunctions among children with COVID-19 showed that the pooled prevalence of smell dysfunction among children with COVID-19 was 15.97% (95% CI: 8.18%–23.77%), the pooled prevalence of taste dysfunction among children with COVID-19 was 9.20% (95% CI: 4.25%–14.16%), the pooled prevalence of smell or taste dysfunction among children with COVID-19 was 15.50% (95% CI: 10.30%–20.70%), and the pooled prevalence of smell and taste dysfunction among children with COVID-19 was 20.21% (95% CI: 14.14%–26.28%) (18). In post-epidemic era, our study showed that pediatric anosmia and dysgeusia are relatively uncommon; 6.43% and 13.34% of children experienced smell and taste disturbances, respectively. However, the prevalence in our study may be overestimated because of the survey mechanism. In adults (or mostly adults), Omicron causes much less dysosmia than previous variants (4–10-fold less) (22). The same is likely true for children, but there are no studies yet to prove that. Since the potential bias due to the survey mechanism and our data may be biased toward higher prevalence, it was not possible to arrive at any firm conclusions about whether Omicron also leads to lower prevalence of chemosensory dysfunction in children.

The pediatric anosmia and dysgeusia from COVID-19 infection typically resolve within 1 week, although in some cases, symptoms persist longer. Based on the results of the univariate and multivariate analysis, older age, cough, headache, sore throat, and asthenia were identified as olfactory dysfunction risk factors, and older age, headache, sore throat, and asthenia were identified as dysgeusia risk factors. It showed that taste and smell dysfunction mainly occurred in older children with severe symptoms. We did not observe that the prevalence of olfaction and gustation dysfunction was significantly affected by the vaccination rate. These findings have been similar to the results of previous studies, the increased incidence of Omicron variant breakthrough infections, and reduction in the protective effect of the vaccine (23, 24). Previous studies have indicated that allergic rhinitis is a protective factor in patients with COVID-19 (25, 26). Based on our research, the rate of allergic rhinitis was lower in the loss of sense of smell or taste group than the no loss of sense of smell or taste group, but the difference was not statistically significant, and further assays need to be performed to validate these observations.

The mechanism of the impairment of olfaction and gustation dysfunction in COVID-19 remained unclear, but some hypotheses have been reported. During the first year of the pandemic, many hypotheses have been made: congestion of the nasal mucosa due to swelling and obstruction of the olfactory cleft, infection and death of receptor neurons, viral neuroinvasion along the nerve, altered and neuronal function due to cytokine release and inflammation (27, 28). However, most of the above-listed hypotheses turned out to be implausible, for various reasons (27). Among the current hypotheses, the most plausible one involves the death of infected support cells in the olfactory epithelium and, as a result, temporary disruption of olfactory receptor neuron function (27).

Our study had several limitations. First, our study was not carrying out objective smell, taste, and hearing tests by objective assessment methods. Second, our study was a cross-sectional online questionnaire study, and respondents mainly had mild to moderate disease, which limits the generalizability of the study findings of this particular population to severe patients. Third, the survey mechanism likely introduces bias, because patients (and their caregivers) are more likely to be motivated and to respond when they have more severe symptoms. Fourth, if the spectrum of respondents is not representative of all infected cases, including a similar proportion of asymptomatic cases, then the prevalence of symptoms will be biased toward higher values.

## 5. Conclusions

In the post-epidemic era, due to the weakening of the pathogenicity of the subvariant of Omicron, the overall condition of children with COVID-19 was mild, the incidence of olfactory and taste disorders was low, the recovery was faster, and the prognosis was better. Cough, runny/stuffy nose, and sore throat are the most frequently reported symptoms in our study. The prevalence of taste and smell dysfunction increases as age increases, and it mainly occurred in older children with severe symptoms.

## Data Availability

The original contributions presented in the study are included in the article/Supplementary Material, and further inquiries can be directed to the corresponding author.
